# The definition and classification of hydrocephalus: a personal recommendation to stimulate debate

**DOI:** 10.1186/1743-8454-5-2

**Published:** 2008-01-22

**Authors:** Harold L Rekate

**Affiliations:** 1 Pediatric Neurosciences, Barrow Neurological Institute, St. Joseph's Hospital and Medical Center, Phoenix, AZ 85013, USA

## Abstract

The aim of this review is to refine the definition and classification of hydrocephalus as a preview to developing an international consensus on the nomenclature of this complex condition. This proposed definition and classification is based on my own work in this area and is intended to promote a debate on the concepts presented.

A literature review of contemporary definitions and classifications of hydrocephalus, and of the historic context in which these concepts developed, is presented. Based on new technology and understanding of hydrocephalus, the rationale for nomenclature is also discussed.

Currently, there is no recognized definition of hydrocephalus. The failure to agree on a working definition impedes progress in understanding the pathophysiology and treatment of hydrocephalus. There are many proposed classifications, each with its own starting point in terms of the definition of the condition. This author recommends that the following definition be used as a starting point to develop a consensus statement defining hydrocephalus: "Hydrocephalus is an active distension of the ventricular system of the brain resulting from inadequate passage of cerebrospinal fluid from its point of production within the cerebral ventricles to its point of absorption into the systemic circulation." Such a definition can be used to develop a rational classification consistent with observations from contemporary neuroimaging and can lead to testable hypotheses. It is concluded that hydrocephalus is a complicated neurologic disorder with many causes and methods of treatment. Clinicians and basic scientists must agree on a working definition of the condition to be able to interpret results from different investigators. Reaching a consensus on a working definition and functional classification should be a high priority for researchers in this field.

## Review

### Background

An electronic search of the medical literature using a National Library of Medicine database (PubMed) using the words "hydrocephalus" and "definition" yielded 77 titles. Only five of these articles actually attempted to define hydrocephalus. The concepts on which these definitions were based were disparate and these potential definitions shared few commonalities [1-8].

In an attempt to be as inclusive as possible, Raimondi [7] defined cerebrospinal fluid (CSF) as all fluid within the intracranial compartment except blood. With that starting point, hydrocephalus was defined as any increase in CSF within the intracranial compartment, including edema and hydrocephalus *ex vacuo*. This straightforward definition allowed many pathologies not universally recognized as hydrocephalus by researchers, such as vasogenic and cytotoxic edema, to be incorporated into CSF pathophysiology. This attempted definition did not gain widespread acceptance, partially due to its failure to lead to changes in understanding or treatment of the conditions that were included in the definition.

In an extensive study supported by the Ministry of Health and Welfare of Japan, Mori and colleagues reviewed a database of 1450 patients with hydrocephalus to develop a classification that would allow outcome to be predicted in individual cases. They excluded tumoral hydrocephalus and defined eight subtypes of hydrocephalus in relationship to the time of onset and severity of brain malformation or injury. They proposed that one of the diagnoses should be "intractable hydrocephalus." They submitted criteria for this tragic condition for which treatment is futile. The general results of the study were that hydrocephalus is not a disease but a symptom or sign that relates to CSF dynamics [3,4].

Finally, Johnston and Teo [2] reviewed the basic mechanism of CSF dynamics in a large variety of conditions, to define where further research is needed and to clarify the pathophysiologic mechanisms underlying these specific problems. This approach demonstrated the great value in directing research efforts to solve the remaining problems. That is also the approach of this review. Table 1 shows a comparison of the published definitions and classifications of hydrocephalus. The goal of each of these authors was different and resulted in significantly different approaches to the definition and classification as reflected here.

**Table 1:** Classifications of hydrocephalus

**Author**	**Concept**	**Controversial areas**
Raimondi [7]	All intracranial fluids except blood found in excess	Includes vasogenic and cytotoxic edema Includes brain atrophy and hydrocephalus *ex vacuo*
Mori [4]	Definition of intractable hydrocephalus	In which case is intervention futile?
Johnston [2]	Includes all abnormalities of CSF pressure and volume	Includes arrested hydrocephalus and cysts
Beni-Adani [1]	Obstructive vs communicating forms limited to infants and small children	Classification devised to define babies who may be candidates for third ventriculostomy
Oi [5]	Classification of infantile and fetal hydrocephalus based on CNS developmental state	Prognosis is dependent on what is occurring at the time the pathology develops. May be a rationale for in utero surgery
Rekate [24]	Based on point of obstruction and developed on a mathematical model	Assumes all hydrocephalus is obstructive

From the above discussion, it is clear that despite numerous "consensus conferences" on the subject of hydrocephalus, there is no consensus on the definition of this problem. The goal of this article is to propose a "straw man" definition to serve as a starting point for a discussion for future meetings so that clinicians and basic scientists can develop a common language. The ultimate goal would be to motivate investigators to work collaboratively toward improvements in treatment paradigms for hydrocephalus.

#### A modest definition

As a starting point for future discussions, I propose the following definition of hydrocephalus: "Hydrocephalus is an active distension of the ventricular system of the brain resulting from inadequate passage of CSF from its point of production within the cerebral ventricles to its point of absorption into the systemic circulation." The elements of this definition are simple and therefore, the number of processes included within it is limited and manageable. Hydrocephalus is an active condition. It is a process that can be demonstrated on neuroimaging studies, and the definition suggests that there is a common underlying cause for its different manifestations: a mismatch between CSF production and its absorption.

Because the definition requires an active process, it excludes brain atrophy or *ex vacuo *hydrocephalus. Similarly, because this definition requires ventricular distention (ventriculomegaly), it excludes conditions in which there is a failure of CSF absorption such as pseudotumor cerebri (also called benign intracranial hypertension) and normal volume hydrocephalus, this latter being limited to patients shunted during infancy, but found to have increased intracranial pressure (ICP) without ventricular distension at the time of shunt failure [9]. The exclusion of these two conditions will provoke especially energetic debate. It is clear that they are close relatives of hydrocephalus as they are associated with high intracranial pressure and are caused by an increase in the resistance to flow of CSF. They are excluded from the above definition because of the absence of ventricular dilatation. This is a point for general discussion.

The definition does not require an increased ICP even though that condition usually co-exists. Therefore, the conditions of idiopathic and secondary normal pressure hydrocephalus (NPH) are included. Acceptance of this definition, or one that incorporates these concepts, offers researchers and clinicians a common language and the opportunity to develop a useful classification system, which should be the goal of a future consensus conference.

The proposed definition requires no specific pathologic process. It does not require a researcher to abandon commitment to a bulk flow concept of CSF dynamics or a concept of pulsatility. It also does not require a commitment to an ability to define how CSF is produced, how it is absorbed, or where it is absorbed. It is a point of departure that allows studies on basic aspects of pathophysiology to be incorporated into the definition.

#### Classification of hydrocephalus: historical aspects

Research into the pathophysiology and treatment of hydrocephalus began with the work of Dandy and Blackfan in the early decades of the twentieth century [10]. At that time little was known about the pathophysiology much less about the treatment of hydrocephalus. Plain radiography and lumbar puncture were the only studies that could be performed on hydrocephalic patients or experimental animals. Late in his career, Dandy was influential in the development of pneumoventriculography, which first allowed neuroanatomic features to be defined in living patients and animal models.

Initially, these investigators were limited to studying hydrocephalus using ventricular puncture and lumbar puncture. With these tools, Dandy and his pediatrician colleague, Blackfan, performed ventricular punctures and injected supravital dyes into ventricles and later performed lumbar punctures. If the dye was recovered in the spinal tap, the hydrocephalus was classified as "communicating." If no dye was recovered in the lumbar theca, the hydrocephalus was considered "obstructive" or "non-communicating." Dandy also performed experiments that defined the choroid plexus as the source of the production of CSF [10,11].

Based on these experiments and on clinical observations, Dandy and later investigators developed techniques designed to treat or at least to ameliorate hydrocephalus, which at that time was essentially a death sentence. Dandy and others attempted to perform internal bypasses called third ventriculostomies via an open craniotomy or endoscopically via a cystoscope or choroid plexectomy. In general, all of these techniques were unsuccessful due to the severity of the hydrocephalus diagnosed in those times.

This classification of communicating or obstructive (non-communicating) was extremely useful for understanding hydrocephalus as well as for guiding the search for therapeutic options for the management of the condition. Soon, however, the classification was recognized as an inadequate portrayal of the pathophysiology underlying hydrocephalus. In a brilliant but infrequently cited study, Ransohoff and colleagues reviewed and updated Dandy's ideas [12]. These researchers realized that the crude techniques available to Dandy were inadequate to understand the spectrum of diseases that led to hydrocephalus. They updated the classification to incorporate what was then understood of the pathophysiology of the condition and proposed that Dandy's classification be modified.

Ransohoff *et al *postulated that there were a variety of potential sites of obstruction to the flow of CSF and thought that all hydrocephalus was obstructive [12]. Using the same criteria that Dandy had used, Ransohoff's group agreed that patients had obstructive hydrocephalus when dye in the ventricles did not communicate with the lumbar theca. However, they believed that the point of obstruction was at the aqueduct of Sylvius or at the outlet foramina of the fourth ventricle. They suggested that this form of hydrocephalus be called "intraventricular obstructive" hydrocephalus. They still believed that hydrocephalus associated with CSF flow from the ventricular system to the lumbar theca was an obstructive process involving scarring of the cortical subarachnoid spaces or failure of the terminal absorption of CSF, presumably, by the arachnoid villi. They proposed that this condition should be called "extraventricular obstructive" hydrocephalus [12].

This nomenclature was useful for a variety of reasons. At that time the only available form of treatment for intraventricular obstructive hydrocephalus involved shunting from the ventricle to the atrium of the heart, ureter, or occasionally to the peritoneum. If the lumbar theca was found to be in communication with the ventricular system, the somewhat safer option of shunting the lumbar theca was possible. At this point, effective surgical treatments became available for the control of hydrocephalus, and the Ransohoff classification was useful in selecting from the several treatment paradigms.

In 1973 the first computerized tomography (CT) scanner was installed in the United States. For the first time, noninvasive techniques were available to define the point of obstruction in hydrocephalus. In the early 1980s magnetic resonance imaging (MRI) became available and provided improved resolution to define the actual point of obstruction of CSF flow that led to hydrocephalus. Hydrocephalus progressed from two types, as defined by the techniques available to Dandy, to multiple finite types that could be defined and treated by specific techniques.

#### The CSF pathway as a circuit diagram

**Figure 1:**
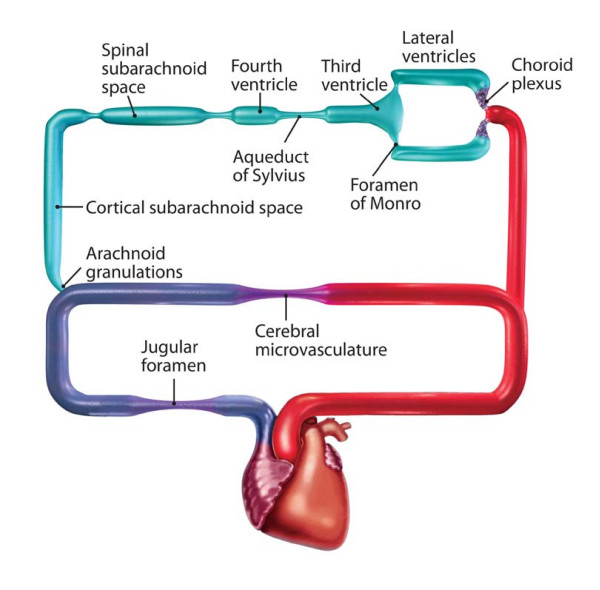
Intracranial hydrodynamics represented as a circuit diagram with a parallel pathway of CSF flow and cerebral blood flow. *With permission from Barrow Neurological Institute*.

Colleagues within the School of Engineering at Case Western Reserve and I developed a mathematical model of the intracranial dynamics of CSF based on a hydraulic analog of Ohm's Law of electrical circuits (Fig. 1). We attempted to measure inherent resistances in this model and mostly were unable to measure pressure differentials (resistance) within the CSF pathways [13,14]. Transducers were inserted within the subarachnoid space, parenchyma, each ventricle, and superior sagittal sinus in normal and hydrocephalic animals and artificial CSF was infused into the various ventricles. We measured the pressure in each compartment simultaneously. Regardless of how ICP was perturbed, we failed to document pressure differentials (resistance elements) within the CSF pathways except in two instances. We always found significant pressure differentials between the cortical subarachnoid space, which had the same pressure as the lateral ventricles, and the superior sagittal sinus [15]. The pressure differentials between the right and left lateral ventricle was about 2 mm Hg when the drained ventricle was small and the septum pellucidum was intact [16].

There are two potential explanations for the failure to measure pressure differentials elsewhere in the system. Firstly, most transducers available for these studies are only accurate to ± 1 mm Hg. Given that the bulk flow of CSF is 0.33 ml/min, we could have failed to detect these pressure differentials if they were lower than the threshold of our instrumentation. To overcome this problem, we infused artificial CSF into the various components. However, we were still unable to measure pressure differentials. The second possibility, which we prefer as an explanation, relates to the viscoelastic properties of the brain itself. The brain has the potential to change its shape and orientation. As soon as a pressure gradient is produced, the brain likely shifts from a point of higher pressure to one of lower pressure. This scenario would explain our inability to measure ventricular-subarachnoid pressure gradients even when CSF flow was obstructed completely.

Each point of potential obstruction (Fig. 1) represents a specific subtype or class of hydrocephalus. This model offers a possible substrate for a useful classification of hydrocephalus based on the point of obstruction, which is possible to define based on contemporary neuroimaging [14,17].

#### Contemporary classification based on neuroimaging

With the rare exception of hydrocephalus associated with overproduction of CSF in patients with choroid plexus papillomas (CPPs), all hydrocephalus is basically obstructive. That the rare CPP causes hydrocephalus is not debated, but why it does so is the subject of some discussion. CPPs are known to lead to increases in the rate of CSF production and are known to cause hydrocephalus.

Normal CSF absorptive mechanisms can clear the amount of spinal fluid produced in the ventricular system at extremely high rates without producing ventriculomegaly. If CSF production and ICP increase substantially, ventricular size increases [18]. When CSF flow is obstructed in the context of increased CSF production, there is a great tendency for ventriculomegaly or hydrocephalus to develop. CPPs, in themselves, can create the only pure form of "communicating" hydrocephalus. However, that these tumors tend to be large and to restrict CSF flow through the foramen of Monro or aqueduct of Sylvius, is more likely to account for the severity of hydrocephalus in this context [18].

#### Proposed classification based on defined points of obstruction

When hydrocephalus is severe, especially in the very young, it may not be possible to define the point of CSF obstruction without introducing tracers into the CSF pathways. In patients treated early in life whose ventricles have become smaller with treatment, it is possible to determine the first site of obstruction to CSF flow on MRI or CT.

**Figure 2:**
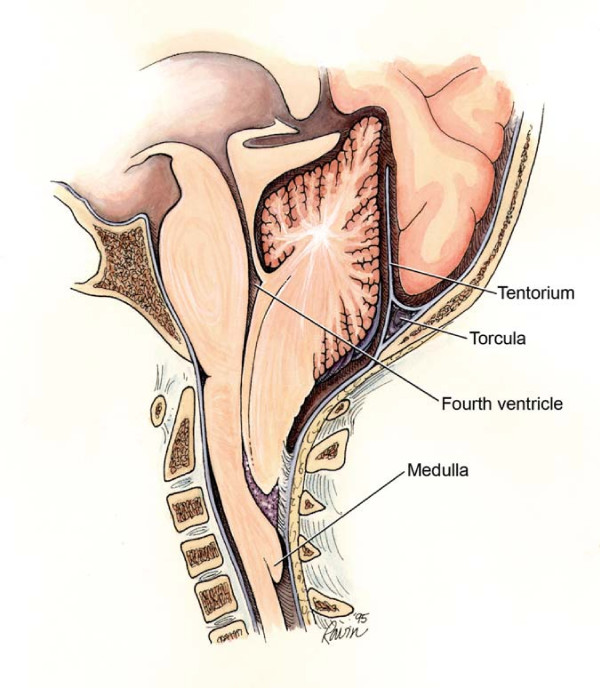
Artist's representation of a Chiari II malformation showing the points of potential obstruction that yield different subtypes of hydrocephalus. *With permission from Barrow Neurological Institute*.

Patients with complex congenital anomalies such as hydrocephalus related to a Chiari II malformation and spina bifida often have multiple sites of obstruction (Fig. 2) [17,19]. It may not be possible to predict a second or downstream point of obstruction. In these patients, only one point may be obstructed or all of these sites may be restricted.

**Figure 3:**
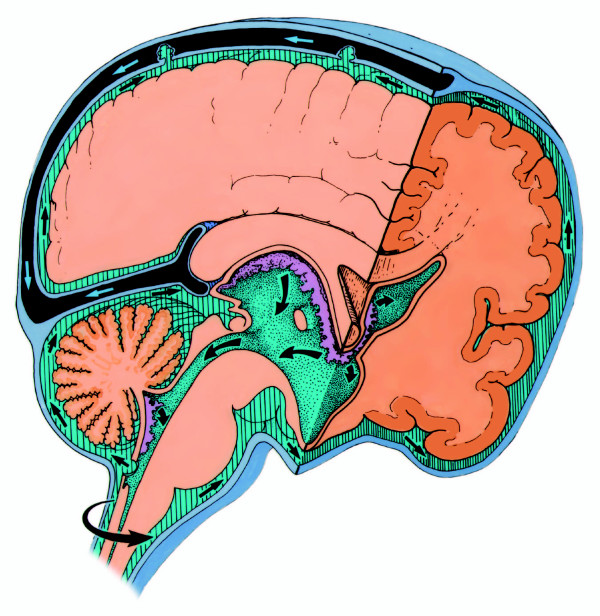
Rationale for endoscopic third ventriculostomy in the case of communicating hydrocephalus. Anatomy of normal CSF pathways. *With permission from Barrow Neurological Institute*.

**Figure 4:**
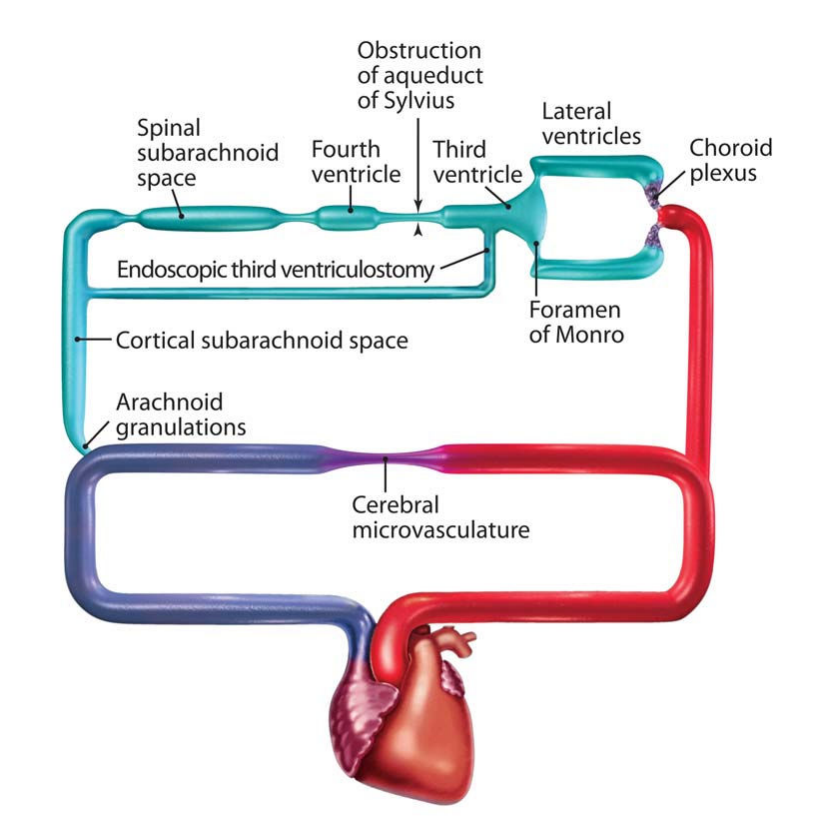
Circuit diagram showing the actual result of an endoscopic third ventriculostomy performed as a bypass between the third ventricle and, in terms of circuitry, the very distal cortical subarachnoid space. *With permission from Barrow Neurological Institute*.

Based on the analyses from our mathematical modeling, our work on the circuitry of CSF flow, and these potential sites of obstruction, I propose the classification shown in Table 2. The table summarizes the types of hydrocephalus and partially lists their potential origins as a starting point for discussion. Table 2 also demonstrates the value of considering hydrocephalus as a derangement of a circuit. It is generally assumed that endoscopic third ventriculostomy (ETV) is only effective for treating obstructive hydrocephalus, and many assume that obstructive hydrocephalus is synonymous with aqueductal stenosis. The growing number of reports on the efficacy of ETV for treating "communicating hydrocephalus" has generated considerable consternation [20]. However, a review of Figures 3 and 4 and the treatment options defined in Table 2 shows that ETV is actually a bypass that avoids obstruction not only in the aqueduct of Sylvius, but also at the outlet foramina of the fourth ventricle and at the basal cisterns at the level of the foramen magnum.

**Table 2:** Proposed classification of hydrocephalus

**Site of Obstruction**	**Pathology**	**Treatment**
None	Choroid plexus papilloma	Removal
Foramen of Monro	Tumor, congenital anomaly, postshunt ventricular asymmetry	Tumor removal, septum pellucidotomy, ventricle shunt
Aqueduct of Sylvius	Congenital lesion, tumor secondary to extraventricular obstruction	ETV, ventricular shunting
Outlets of fourth ventricle	Chronic meningitis, Chiari II malformation	ETV, ventricular shunting
Basal cisterns	Meningitis, post subarachnoid hemorrhage	ETV, ventricle shunt, spinal thecal shunt
Arachnoid granulations	Hemorrhage or infection in infancy	Ventricle or thecal shunt
Venous outflow	Skull base anomalies, congenital heart disease	Ventricle or thecal shunt, treatment of vascular anomaly if possible

ETV: endoscopic third ventriculostomy

#### Alternative classifications

The above discussion is one way of analyzing hydrocephalus and its classification. It meets the requirements for effective classification as set forth above, improves understanding of the condition, and points to areas that can be improved by ongoing research efforts. Nonetheless, other potential classifications, including those based on age, underlying cause of hydrocephalus, or neurologic outcomes, need to be analyzed. There has been considerable interest in the possibility that hydrocephalus may develop as a result of pulsatility causing ventricular dilatation [21,22]. This aspect of hydrocephalus and the intrinsic viscoelastic properties of the brain relate to the different responses among patients with the same anatomic lesions and degree of obstruction [23]. In the future, this aspect of hydrocephalus may need to be included in a comprehensive classification scheme.

## Conclusion

Obtaining a consensus on a working definition of hydrocephalus and especially on a method of classifying this complicated condition is a challenge worth pursuing. A consensus would improve the focus on basic research, the development of logical approaches to treatment decisions, the planning of prospective trials, and the development of new technologies to improve the outcomes of this most chronic of medical conditions.

## Competing interests

HLR has a consulting agreement with Codman Corporation, a Johnson and Johnson company, to assist in the development of shunt systems.
